# Fabrication of a Lab-on-Chip Device Using Material Extrusion (3D Printing) and Demonstration via Malaria-Ab ELISA

**DOI:** 10.3390/mi9010027

**Published:** 2018-01-14

**Authors:** Maria Bauer, Lawrence Kulinsky

**Affiliations:** Department of Mechanical and Aerospace Engineering, University of California, Irvine, CA 92697, USA; mebauer@uci.edu

**Keywords:** 3D printing, fused deposition modeling (FDM), material extrusion, leakage control, malaria, enzyme-linked immunosorbent assay (ELISA), extreme point of care

## Abstract

Additive manufacturing, such as fused deposition modeling (FDM), has been increasingly employed to produce microfluidic platforms due to ease of use, wide distribution of affordable 3D printers and relatively inexpensive materials for printing. In this work, we discuss fabrication and testing of an FDM-printed fully automated colorimetric enzyme-linked immunosorbent assay (ELISA) designed to detect malaria. The detection platform consists of a disposable 3D-printed fluidic cartridge (with elastomeric silicone domes on top of reagent-storage reservoirs) and a nondisposable frame with servomotors and electronic controls such as an Arduino board and a rechargeable battery. The system is controlled by a novel interface where a music file (so-called “song”) is sent to the Arduino board, where the onboard program converts the set of frequencies into action of individual servomotors to rotate their arms a certain amount, thus depressing specific elastomeric domes atop reagent reservoirs and displacing the specific reagents into the detection wells, where bioassay steps are executed. Another of the distinguished characteristics of the demonstrated system is its ability to aspirate the fluid from the detection wells into the waste reservoir. Therefore, the demonstrated automated platform has the ability to execute even the most complex multi-step assays where dilution and multiple washes are required. Optimization of 3D-printer settings and ways to control leakages typical of FDM-printed fluidic systems are also discussed.

## 1. Introduction

In developing countries, infectious diseases pose a heavy burden to the population, including loss of life and economic hardship [1,2]. The problems infectious diseases pose to developing countries are exacerbated by lack of affordable diagnostic platforms that can offer a rapid detection for malaria and other deadly infections [2,3]. 

For the case of malaria, the region with the highest number of infections (88%) and deaths (90%) is sub-Saharan Africa. According to the World Health Organization (WHO), over 200 million people were infected by malaria in 2015, of which 438,000 people died [4]. To improve the quality of malaria management, while not mistreating other infections, the WHO recommends positive diagnosis of malaria prior to initiating malaria treatment [5]. This is especially important due to a very low specificity (0–9%) of clinical finding-based diagnosis for children in Africa, and a high malaria over-diagnosis of 30% shown during an evaluation of the integrated management of childhood illness (IMCI) clinical algorithm [6].

Despite the urgent need for diagnostics in developing countries, many complex tests that are routinely used in developed countries are often not appropriate or available. Standard diagnostics for malaria with high specificity and sensitivity are polymerase chain reaction (PCR)- or loop-mediated isothermal amplification (LAMP)-based [7]. However, these complicated tests require intensive laboratory equipment, well-trained personnel and controlled environmental settings. Rural settings in developing countries lack not only equipment and trained personnel, but also access to running water and reliable electrical services [2,8]. Supplementing complex laboratory tests is the recently emerging field of rapid, inexpensive and user-friendly point-of-care (POC) tests [9]. POC tests used for malaria include microscopy and laminar flow-based rapid diagnostic tests (RDTs).

Microscopic diagnosis is based on examination of stained blood films using Giemsa’s, Wright’s or Field’s stains. The lower limit of detection depends heavily on the expertise of the microscopist, and varies from 5 parasites/µL for experts to 50–100 parasites/µL for average examiners. This can lead to false diagnosis, especially in the case of low parasite count or asymptotic malaria [10]. Also, limited access to good microscopy and electricity, patient load, epidemiology of malaria and availability of trained technicians might not allow for use of microscopy and only leave RDTs as a viable detection option [5].

Today, 86 malaria RDTs are available [6], all consisting of capillary flow membranes containing specific antimalaria antibodies to detect malaria antigen, foremost *P. falciparum*-specific protein in patients’ blood. Detection times can be as low as 15 min with sensitivity for *P. falciparum* of 99.7% and a specificity of 94.2% [6]. The main disadvantage of RDTs for malaria is the low sensitivity to non-falciparum infections. Moreover, RDTs are limited to single-step assays, reduce precision by inaccurate sample volume, limit sensitivity due to total test volume, lack possibility for response enhancement (enzyme reaction), require good antibody preparation, vary in analysis time depending on sample properties (for example, viscosity), may have obstructions in matrix components, and require sample pretreatment for non-fluid samples [11].

Strengths and weaknesses of different diagnostic approaches for malaria detection are summarized in Table 1.

In this work, we present an alternative diagnostic device for POC, which can be 3D-printed on low-cost printers in remote hospitals or health kiosks, and allows for automation of colorimetric testing. The key advantages of this approach are the ease in distribution of print files and programs for various infectious diseases via the internet, low cost of platform components such as servomotors, and ubiquitous presence of smartphones that can serve as controlling and detecting elements of the presented malaria-detection platform. Furthermore, as reagents can be stored in hospitals and loaded when needed, no cold storage chain for the device is necessary. The presented device can be applied to complex multistep bioassays that require multiple wash/dilution steps and are thus inappropriate for lateral-flow assays.

## 2. Background

Lab-on-chip (LOC) platforms containing fluidic networks of microchannels are often utilized for POC testing. These LOC platforms are highly portable, while integrating all necessary reagents to substitute a set of bulky and expensive laboratory equipment [21]. This miniaturization further leads to additional cost-reduction of tests by eliminating the need for laboratory settings and trained personnel, as well as by a drastic reduction in reagent consumption [22].

Haeberle and Zengerle [23] characterize microfluidic platforms (LOC devices) into capillary-driven systems (RDTs), “microfluidic large-scale integration” systems, electrokinetic platforms, centrifugal microfluidics, and “free scalable non-contact dispensing” devices. The main functions integrated on these platforms are: pumping and valving, mixing, separation, reagent storage and sample preparation [24]. A 2013 market review of LOC applications showed blood glucose analysis, electrolytes analysis, HIV diagnostics and determination of cardiac markers as some of the main applications of LOC systems by leading companies such as Abbott, Alere, Arkray, Bayer, LifeScan, Menarini Diagnostics, Roche and Siemens [24]. Diagnostic tests that run on microfluidic devices range from immunoassays (based on immune responses to an antigen of a particular pathogen) to molecular diagnostics (detecting and oftentimes amplifying deoxyribose nucleic acid (DNA) or ribose nucleic acid (RNA)) [25]. While both diagnostics have been implemented on microfluidic devices, implementation of molecular diagnostics is generally more challenging due to more complex assay steps. For example, the use of polymerase chain reaction (PCR) for amplification of nucleic acids requires elaborate sample preparation and complex thermal cycling [25]. The following section will review some microfluidic devices for POC.

### 2.1. LOC Devices for POC Based on Molecular Detection and Immunoassays

Liao et al. [26] implemented POC testing for HSV-2 virus on a portable molecular diagnostic device. The so-called “smart cup” combines capillary fluidics and isothermal amplification to carry out a quantitative fluorescent LAMP assay. The design uses the smartphone flashlight to excite the fluorescent dye and the smartphone camera for quantitative readout. Although the heating technique and the readout are very economic (Mg–Fe pouches are only $0.15), the design uses a custom-made microfluidic chip consisting of Qiagen silica membrane as entry port, and solvent-bonded, milled polymethyl methacrylate (PMMA) layers containing the microfluidic structures as described in Liu et al. [26,27].

In another publication by the same group [28], two custom-made, layered microfluidic chips were used for plasma separation and detection of nucleic acids via reverse-transcriptase LAMP. The plasma separation chip consisted of machined PMMA, plasma-separation membranes, and double-sided and single-sided adhesives. Another microfluidic chip was used for nucleic acid extraction and amplification [26,27,28]. The sample-to-answer testing would include loading the sample into the first chip, transfer of the plasma to the second chip as well as manual placing of the second chip onto a heating platform [28]. Besides a number of manual steps in running this assay, the fabrication sequence to produce these platforms is rather involved. 

Microfluidic chips produced via injection molding can present a faster fabrication route and could also be a relatively affordable option for production of large numbers of fluidic chips. This fabrication option for enzyme-linked immunosorbent assay (ELISA) LOCs is discussed by Chin et al. [29]. Injection-molded LOCs were used to run hundreds of samples in Rwanda to detect HIV and syphilis simultaneously from 1 μL of whole blood. While injection molding typically is used to produce features above 100 μm in size, tight control of process parameters allowed for reduction of feature sizes to 1 μm [29]. The materials used to form the microfluidic chips are polystyrene and cyclic olefin copolymer. Cost and time per chip of $0.10 and 40 s, respectively [29], render chip fabrication via injection molding a low-cost and high-throughput manufacturing technique. While highly desirable if large quantities of identical parts are produced, injection molding is not an appropriate fabrication approach for prototyping or production of a limited number of parts, due to high cost of molds.

### 2.2. 3D Printing for LOC Devices

Most LOC devices are based on microfabrication methods using materials such as glass, plastic or polydimethylsiloxane (PDMS) [30]. While these fabrication methods require access to highly specialized and expensive microfabrication tools, as well as fabrication of a master for replica molding [31,32], recent developments in 3D printing, such as emergence of a wider range of materials and inexpensive printers, open new possibilities for rapid fabrication of affordable highly customizable LOC platforms [31,33,34,35]. 

For example, a POC device that implements LAMP for genomic detection of *Escherichia Coli* and *Staphylococcus Aureus* was fabricated with 3D-printing technology by Stedtfeld et al. [36]. The overall structure of the device was manufactured using stereolithography (3D printing based on curing liquid photosensitive resin by UV light [32]), while a channel network on the microfluidic chip was obtained using rubber-assisted hot embossing of polyester film with a 3D-printed mold. The polyester film was then assembled with a hydrophobic membrane and closed off with patterned adhesive [36]. The developed device is operated through an iPod Touch, which is also utilized for data analysis and Wi-Fi connectivity. A sample is loaded manually using a pipettor, while hydrophobic membranes and manual taping of vents after fill maintain fluid in the channels and free of contamination. [36].

Material jetting is another 3D-printing technology, and was used by Erkal et al. [37] to create a microfluidic device which holds electrodes for electrochemical detection of dopamine and nitric oxide. Another 3D-printed fluidic device demonstrated by the same group was used to measure the presence of adenosine triphosphate (ATP) while observing oxygen stimulus concentration in the sample [37]. Both devices were fabricated via an Objet Connex 350 multi-material printer.

Stereolithography and material jetting are well suited for designs requiring leakage-free bonding between adjacent layers, but these technologies are significantly more expensive than alternative 3D technologies such as fused deposition modeling (FDM) [31]. Prices for low-end FDM printers recently dropped from $14,000 to $300 due to expiration of patents, and are expected to decrease further as a large community of hobbyists has evolved around 3D printing, making hardware open source and sharing designs and ideas online [38]. 

**Figure 1:**
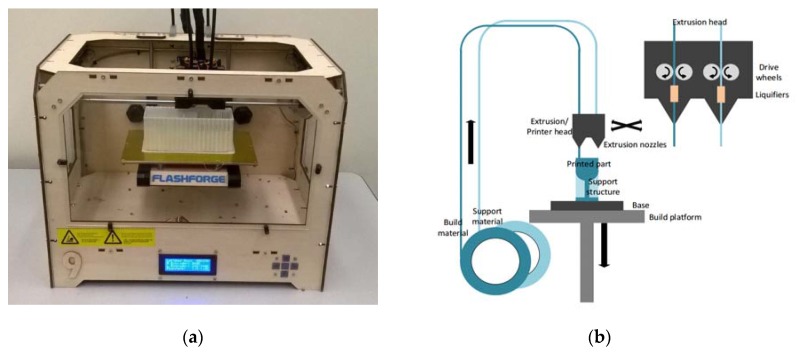
(**a**) Flashforge Original Creator, the fused deposition modeling (FDM) system used for building devices in this work; (**b**) Principle of material extrusion: Thermoplastic filament is heated while extruded through a nozzle onto the build plate. It resolidifies as it cools down, and after the build plate is lowered, the next layer is deposited atop, thus forming the desired geometry.

The principle of FDM is based on layered deposition of liquefied material, and is illustrated in Figure 1. Thermoplastic filaments are stored on spools and extruded through heated nozzles, where the plastic is liquified upon reaching the glass transition temperature. The molten material is then deposited onto the base, where it cools down and solidifies. To deposit a new layer atop of solidified material, the build platform holding the base is lowered. The height of one layer is defined by the lowering distance of the build platform between successive layers, and is as low as 100 µm [39,40,41].

To save material and time, parts are typically not printed solid but filled with a hexagonal honeycomb structure (this so-called infill differs in the various systems and can be of different geometric pattern, such as the square pattern utilized in Airwolf systems). The surface of printed parts consists of the denser shell of material. These shells are made up of about one to four layers (the number can be chosen by the user) of extruded material that give the part its shape and seal the hollow infill.

Waheed et al. [33] recently analyzed advantages and disadvantages of different FDM systems utilized for the fabrication of microfluidic devices, and found that these systems are simple to use and are able to fabricate affordable microfluidic systems. However, FDM-printed parts present a staircase effect deriving from the layered fabrication method. The extent of this effect is impacted by the layer height and negatively affects the surface texture, as well as limits the resolution of printed parts. The average deviation for features printed in the X and Y directions was reported to be 60.8 and 71.5 µm, respectively [33].

Among the nine FDM systems analyzed in Waheed et al. [33] was also the MakerBot Replicator 2X (MakerBot Industries, LLC, New York, NY, USA), which was recently demonstrated by Kadimisetty et al. [42] to be capable of manufacturing a low-cost ELISA platform for the electrochemiluminescent detection of cancer proteins. The channel height of this fluidic chip was 200 µm, which corresponds to the layer height of the MakerBot Replicator 2X [42]. To actuate liquids, Kadimisetty et al. [42] rely on gravity by manually tilting the device.

Another device that uses 3D printing for device manufacturing, as well as a smartphone for readout, is described by Berg et al. [43]. The smartphone is not only used to read out the colorimetric ELISA (supported by a lens in the printed device) from a 96-well plate, but also allows for a custom mobile application with an interactive user interface. To allow for improved data processing, a server was used in conjunction with the device and a smartphone. Smartphones are increasingly used in POC devices [9,26,36,43] to replace bulky and expensive optical instruments, benefitting from portable dimensions, built-in light source and integration of multiple functions such as imaging, image processing and providing a user interface via touchscreen, as well as Bluetooth and Wi-Fi connectivity. Wireless connectivity is especially important for POC applications as it enables one to remotely analyze data, and provides real-time feedback from specialists in central hospitals to patients in remote areas [36].

While all microfluidic devices use some type of force propelling liquid through channels, Iwai et al. implemented a human-powered pumping system, allowing for resource-free (no electricity or external components) actuation of fluids through channels. In this design, a human finger pushes down onto an air-filled pressure chamber exerting increased pressure onto fluids in connected microfluidic channels. As the finger releases the dome on the pressure chamber, membrane-type fluidic diodes close, preventing backflow. Cantilever-based diodes are integrated to allow for multiple refills of the channels from fluid inlets. An injection-molding process was developed for low-cost mass production, however, it required increasing channel dimensions from 100 to 300 µm, and introducing the need for oxygen plasma and thermal treatment for bonding of layers [44]. While the finger-powered microfluidic device does not need external power sources, it is difficult to automate when a sequence of fluidic steps is required.

The device suggested by the authors of this work employs 3D-printed (FDM) microfluidics and actuation of reagents via servomotors, mounted onto a reusable case. For production of these devices, printers could be placed in remote hospitals of developing countries to allow on-demand manufacturing of the devices. Implementing electronics onto a reusable case not only allows one to reduce costs, but also affords higher adaptability of the reusable part to different types of immunoassays. Together with control of electronics and processing readout via a smartphone, the design enables the automation of complicated ELISA procedures with multiple steps, including multiple usages of fluid from the same reservoir, flow reciprocation, repeated emptying of the wells, and various incubation steps. Online distribution of print files as well as of control sequences (Arduino C-files or audio files as presented below), and increasingly ready access to 3D printing and disposable syringes and servomotors, allows for worldwide dissemination and access to an automated bioassay technology that does not need laboratory settings or trained personnel to function.

## 3. Materials and Methods

### 3.1. Principle of Automated Bioassay Platform

The produced automated bioassay platform consists of the 3D-printed fluidic cartridge (with embedded channels and attached silicone domes), as well as a reusable frame holding the servomotors and electrical components. Automation is implemented by an Arduino board controlling servomotors and other components such as microheaters, temperature sensors and LEDs. 

**Figure 2:**
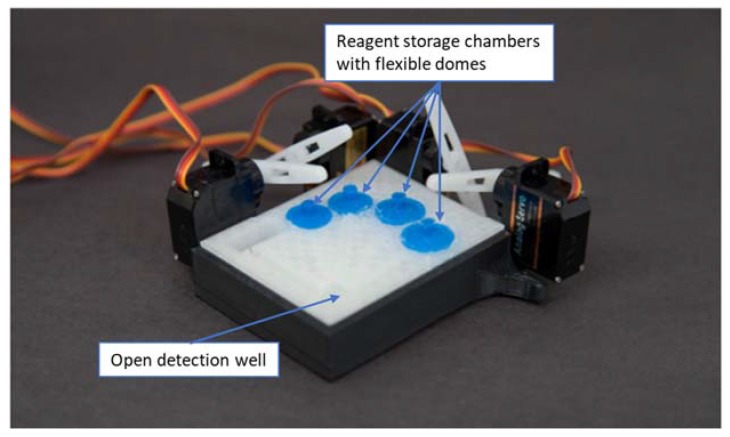
First design moving fluid through enclosed channels by servomotor actuation controlled from a smartphone audio jack.

In the course of this study, three generations of prototypes were fabricated. The first, smallest prototype (with a footprint of 75 × 50 mm), shown in Figure 2, contained four reagent-storage chambers and enclosed channels leading to an open detection/reaction well. Silicone domes were sealed to the cartridge with Sil-Poxy Silicone Adhesive (Smooth-On, Inc., Macungie, PA, USA). The servomotors (TowerPro SG90 Micro Servos, Shenzhen Hao Qi Core Technology Co., Ltd., Shenzhen, China and HS-5645MG Digital High Torque Servo Motor, HITEC RCD USA Inc., Poway, CA, USA) were controlled by a smartphone or laptop (via Arduino board) and plastic arms attached to servomotors pushed on silicone domes to expel the reagents from the storage chambers into the detection/reaction well (micro-servomotors are available for less than $5 online [45]).

**Figure 3:**
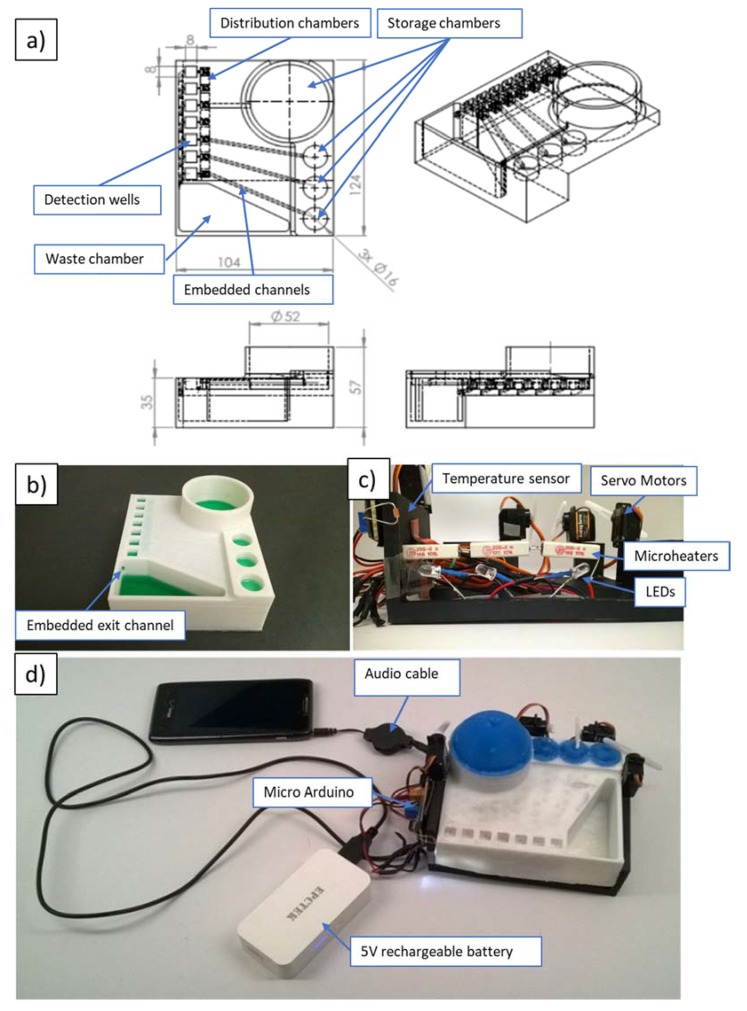
Second design powered by portable battery and controlled via smartphone. The design implements fluid actuation, lighting, incubation (heating and temperature control). (**a**) Major dimensions of prototype in mm; (**b**) Printed cartridge with colored water; (**c**) Reusable frame holding electronics; (**d**) Fully assembled device with electronics connected to portable battery and audio outlet of smartphone.

In order to demonstrate the developed device with a bioassay, the cartridge had to be scaled up to hold the required volumes (as much as 3.6 mL washing buffer) of reagents. The storage reservoirs of the original prototype could hold about 50 µL for stop solution, conjugate solution and tetramethylbenzidine (TMB) substrate, and about 70 µL for washing buffer. Figure 3 shows the second prototype with a footprint of 124 mm × 104 mm. 

Besides increased reagent volumes, this device integrated microheaters and a temperature sensor for incubation, as well as LEDs underneath the detection wells for improved illumination for higher fidelity during readout. Similar to the first prototype, this device was controlled by a microcontroller board for Arduino (OSOYOO Mini USB Nano V3.0 ATMEGA328P, Pintree Electronics Ltd., Richmond, BC, Canada), connected to a smartphone or a laptop. The electronics were powered by a 5-V rechargeable battery. The propulsion of the fluidics followed the same principle of servomotors pushing flexible silicone domes atop of reagent-storage chambers. The second prototype included seven detection/reaction wells that had integrated antigen-coated (for ELISA reaction) plastic test tubes broken apart from the commercial 96-well-plate format colorimetric malaria ELISA bioassay (IBL International GmbH, Hamburg, Germany). All channels from the storage chambers lead to the distribution chambers (a series of interconnected shallow reservoirs), which were designed to allow for equal allocation of the reagents to all detection wells (see Figure 3a). Exiting the detection wells, fluid would flow through the embedded exit into the waste chamber (see Figure 3b).

**Figure 4:**
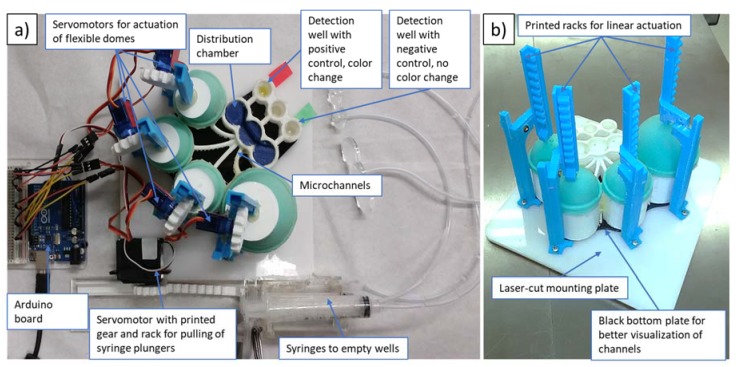
(**a**) Top view of the third prototype incorporating a 3D-printed set of racks, gears and waste syringes (two waste syringes are stacked one atop the other); (**b**) Side view of the third prototype (gears, motors and syringes are removed to facilitate the clear view of the fluidic platform).

Some of the deficiencies of the second prototype included leakage and the possibility of cross-contamination between neighboring detection wells. To address these issues, we have developed a third prototype, shown in Figure 4 (with footprint similar to that of the second prototype). In order to eliminate cross-contamination, the microfluidic network of channels was redesigned to allow for wider separation, while Plasti Dip (Plasti Dip Int., Minneapolis, MN, USA) was used for coatings of the acrylonitrile butadiene styrene (ABS) structure to eliminate leakages. Plasti Dip clear coating was brushed on and cured overnight. For future devices, Plasti Dip coating could be replaced with biocompatible coatings such as liquid silicone rubber (QP1-2XX Liquid Silicone Rubber (LSR), Dow Corning^®^ ,Midland, MI, USA), which is specifically developed and tested for use in medical devices. Additionally, to avoid cross-contamination, the position of the detection wells was lowered with respect to the distribution chambers, preventing backflow. In contrast to previously used rotating arms attached to servomotors (see Figure 2 and Figure 3), we found that linear movement of vertically positioned 3D-printed actuators (see Figure 4b) connected via 3D-printed gears to servomotors was a more reliable way to dispense controlled amounts of reagents by depressing elastic domes of the storage chambers. For qualitative readout, we found that incubation at room temperature was sufficient to clearly distinguish between positive and negative controls without the use of LED lights. Therefore, microheaters, the temperature sensor and LED lights were not utilized in the final prototype of this work. To extend the present platform design for quantitative tests, microheaters and LED light(s) should be integrated into the platform (similarly to our second prototype).

All 3D-printed parts were designed using SolidWorks (Versions 2014/2015 through 2016/2017, Dassault Systems Corp., Vélizy-Villacoublay, France), exported as STL files and converted into g-code using MakerWare 3.4 (MakerBot Industries, New York, NY, USA). All parts were printed using a Flashforge Original Creator (FlashForge Corp., Jinhua, China) with 1.75 mm ABS filament (MatterHackers, Inc., Lake Forest, CA, USA). 

For faster fabrication of the third prototype, the bottom plate (serving as the frame for servomotors) of the reusable case, as well as lids for the wells (to hold emptying tubes in place), were laser-cut from cast acrylic sheets (McMaster-Carr Supply Co., Elmhurst, IL, USA) using an 80 W Speedy 360 Trotec Engraver (Trotec Laser GmbH, Lyss, Switzerland). However, the bottom plate can be replaced by any flat surface that allows for mounting of the servomotors and printed components on the reusable frame. To create flexible domes, 3D-printed molds (printed on Flashforge Original Creator with 1.75 mm ABS filament) were filled with Mold Star 30 elastomer (Smooth-On, Inc.). The print settings that were utilized in the printing of dome molds are given in the Appendix, Table A1. The molds consisted of bottom and top halves with a 2 mm wide gap for the elastomer to form the dome. The molded domes were allowed to cure overnight. 

Reagents were injected into storage chambers with a syringe needle piercing through the elastomeric domes before the use of the device. Utilization of a gauge-21 needle allowed the elastic domes to self-seal after injection of the reagents.

### 3.2. Control of Devices

The servomotors are controlled by an Arduino Uno connected to a smartphone (Lumia 521, Nokia, Espoo, Finland) or laptop (Toshiba Satellite Pro, Toshiba, Tokyo, Japan) and powered either from the laptop or by a rechargeable 5-V battery (EPCTEK^®^ 5200 mAh Power Bank Portable Charger, EPCTEK Technology Co., Limited, Hong Kong, China). The four TowerPro SG90 Micro Servos servomotors (with 90° rotation range) were utilized to push the silicone domes on top of the reagent-storage chambers. Two 10 mL syringes (see Figure 4a) had their plungers pulled out by the set of racks and gears attached to a servomotor (HS-5645MG Digital High Torque Servo Motor with continuous rotation). Pulling out of the plunger provided the necessary suction to empty out the reagents from the detection well through tubes after each washing step.

**Figure 5:**
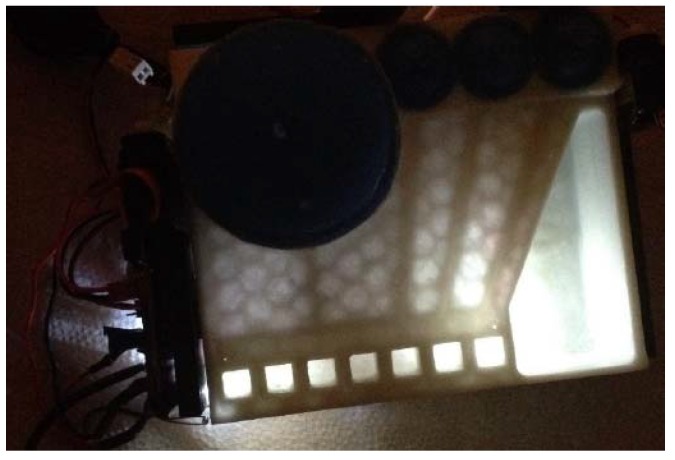
Effect of LEDs for readout in the dark. Empty cartridge.

Incubation at 37 °C was enabled via three wire resistors (5 W, 1 Ω, each 25 mm) under the detection wells, and a heat sensor in close proximity to the detection wells allowed for temperature control. Readout was implemented via taking an image with a smartphone camera and processing that image with the Color Catcher phone app. The Color Catcher application exported the color in the image as RGB values. These values could be read off the phone or sent out via the phone network, Bluetooth or Wi-Fi to other locations (for example, to a hospital laboratory). To support reproducible readout with a smartphone camera, LEDs were positioned under the detection wells and the color was imaged without any additional light source to ensure consistent light settings (see Figure 5).

The automation of the bioassay platform was implemented via a music file (.wav) that contained sounds of different frequencies and duration (an example is given in the Appendix, Table A2). Arduino microcontrollers were programmed using Visual Studio in C++ (see the program in Supplementary Material) to translate music input into specific electrical signals to control electronic components such as servomotors, LEDs and so on. Various frequency ranges controlled different devices. For example, a frequency between 1100 and 1400 Hz actuated servomotor 1, while a frequency between 4100 and 4400 Hz changed the position of servomotor 4, allowing for the control of multiple components by a single music file. Advantage of this approach is that the platform is adaptable to different procedures (e.g., for different bioassays), because the Arduino serves as a translator rather than holding a single program for the platform. Additionally, storage and distribution of the programs is as easy as storing or sending a song, and devices to control the platform range from MP3/CD players to smartphones to laptops. Even a car radio only equipped with a compact audio cassette allows one to run the bioassay. Different websites allow one to generate the sequence of sinusoidal signals utilized for activation of the different functions (e.g., www.wavetones.com and www.onlinetonegenerator.com). These can then be downloaded as wav files. 

### 3.3. ELISA Steps and Implementation

**Figure 6:**
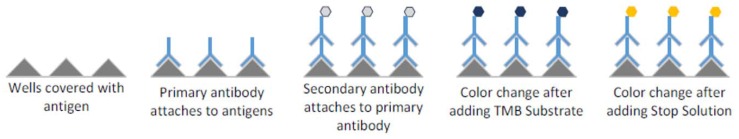
Schematic malaria ELISA steps. Test tubes contained in the malaria kit are precoated with specific antigens. Primary antibodies (present in infected blood due to a patient’s immune reaction to malaria pathogens) bind to antigens. Secondary antibodies (contained in malaria conjugate) are employed for optical detection as they are conjugated to the enzyme, effecting the color change of the solution after TMB substrate is added. The presence or absence of the color change of the solution in the detection well is evaluated after the stop solution is added.

The malaria bioassay (content of the bioassay kit is given in the Appendix, Table A3) implemented in this study is based on antigen–antibody reactions occurring in the test tubes inside the detection wells. The steps are illustrated in Figure 6 and include the attachment of primary antibody (present in infected blood due to an immune reaction to malaria pathogen) to antigen immobilized on the bottom of the test wells, attachment of a secondary antibody (contained in malaria conjugate) to the primary antibody, color change enabled by the enzymes attached to the secondary antibody after addition of TMB substrate, and stopping the color-change reaction with addition of the stop solution. To avoid false reading due to unbound antibodies, multiple washing steps are performed.

Prior to the start of the automated bioassay protocol, precoated mini-vials are broken from the 96-break-apart strips (part of the malaria-Ab kit) and inserted into the cavities to form the detection wells. Subsequently, the positive and negative controls, supplied as part of the kit and representing patients’ samples, are pipetted into these wells. After incubation of the controls in the wells (see Table 2 for details of each step), the automated protocol is started. Automation includes pre-determined sequence of servomotors pushing on flexible domes atop four reagent-storage chambers containing the four reagents (bottom to top in Figure 4a): washing solution, malaria conjugate, TMB substrate and stop solution. When the arm of a servomotor presses on the dome, a liquid reagent is expelled from the reagent storage below the dome to go into the distribution chamber. About 0.20 mL of a reagent is left in the reagent chamber after it is fully compressed.

In order to evaluate distribution of fluids via the distribution chamber, equal allocation of a total volume of 1.9 mL of fluid into the two outer detection wells was tested and results showed an average difference in volumes of 15%, equaling 0.26 mL, with a standard deviation of 0.17 mL. Also, fluid loss due to liquid remaining in the fluidic network, which includes the channels and the distribution chamber, has a mean of 0.24 mL (with standard deviation of 0.19 mL). Therefore, due to a predictable low loss of reagents during the assay, it is possible to compensate for that loss by filling the reagent chambers by about 0.44 mL (0.20 mL of dead volume in the reagent chamber as described above and additional 0.24 mL loss in the fluidic network).

Reagents are sequentially propelled through the channels into the distribution chamber and flow from there into the detection wells. To empty the wells after washing steps, the continuous servomotor mounted on the side of the reusable case (see the bottom of the Figure 4a) pulls the plunger out of the syringes and hence empties the wells through the tubes attached to the syringes. After application of the stop solution (also loaded into one of the domed chambers), a color of the positive control solution changes from blue to yellow. Table 2 summarizes the steps performed on the 3D-printed automated platform (recommended steps including readout on ELISA reader are listed in Appendix, Table A4).

## 4. Results & Discussion

### 4.1. Optimization of FDM Printer Settings

**Figure 7:**
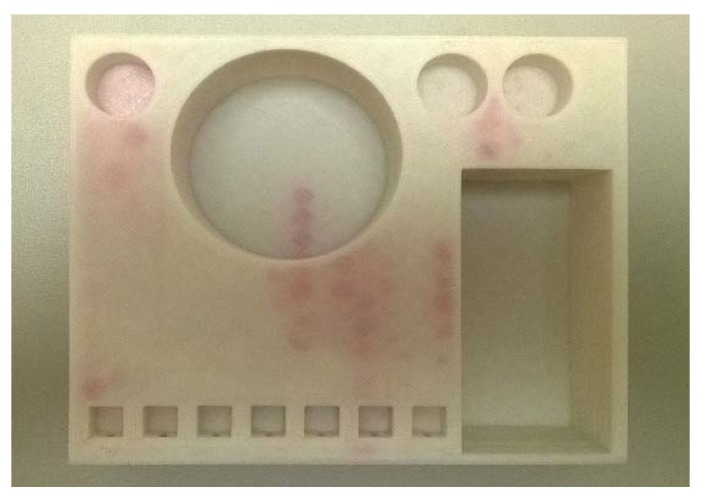
Printed part with leakages showing red. Dyed water marks the hexagonal infill structures filled with water through leakages from chambers and enclosed channels into the part.

Due to imperfect fusing of the adjacent layers, microfluidic devices printed via fused deposition modeling can frequently be subject to leakage [46]. Figure 7 shows a printed part (the second prototype) where stained sections indicate the presence of leaks after water containing the red-colored dye was passed through the device. 

To fabricate leakage-free fluidic cartridges, different print settings were tested and analyzed for their contribution to maintaining the designed device and channel geometry while avoiding leakages. Table 3 summarizes the influence of various printer settings on the quality of the test print. While it is known that in order to decrease leakage, a high infill can be beneficial [46], this option was not considered, as it leads to significant increase in print time and attendant rise in fabrication cost. For testing of impact of different print settings on leakage and geometric accuracy, the following parameters were kept constant throughout all tests: heating platform temperature: 115 °C, number of shells (walls surrounding the infill): 4 (standard setting is 2), and travelling speed: 90 mm/s. Varied settings were: layer height (0.1 mm to 0.2 mm), extrusion speed (60 mm/s and 80 mm/s) as well as extrusion temperature (230 °C, 240 °C, 243 °C (any higher temperature leads to bubbles in extruded material through overheating)). The optimal print setting was found to be a combination of 240 °C extrusion temperature, an extrusion speed of 60 mm/s, and a layer height of 0.16 mm. 

**Figure 8:**
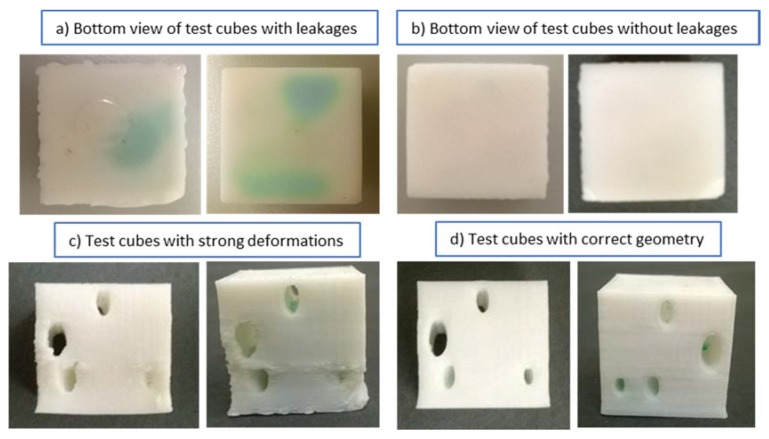
Test cubes with leakage after submerging in dyed water (tests 5 and 10, for settings see Table 2), without leakage (tests 2 and 3), with large deformations (tests 1 and 7) and negligible deformations (tests 5 and 3).

**Figure 9:**
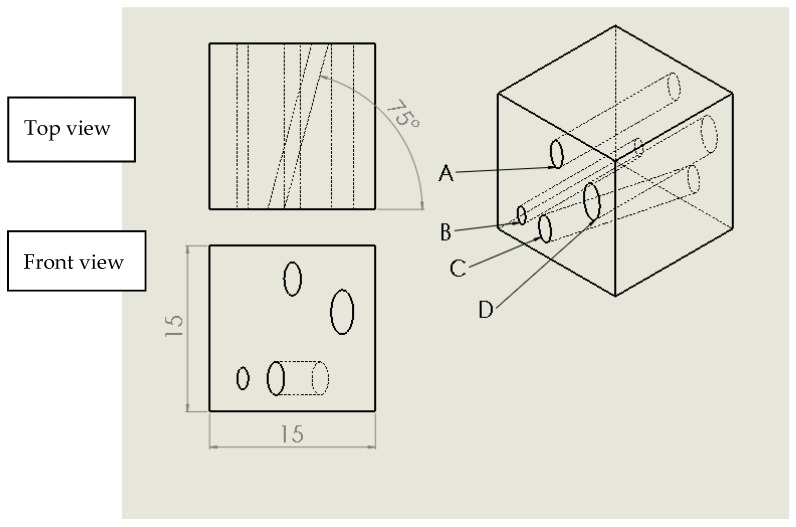
Major dimensions of test cubes in mm. Horizontal channels A, B and D run normal to the sides of the test cube, while channel C is inclined 75° with respect to the side of the cube. Channels have elliptical cross sections (major axis (mm) × minor axis (mm)): A, C: 3 × 1.5, B: 2 × 1, D: 4 × 2.

Figure 8 demonstrates the examples of test cubes with and without leakage after submerging in dyed water (Figure 8a,b), and qualitative rating of geometry (Figure 8c,d). To minimize distortion of embedded fluidic channels as well as to avoid the need to have supporting pillars inside channels, an elliptical cross-section able to support the channel roof was selected. The rhombus (diamond shape) is another channel geometry that is self-supporting [46]. The geometry of the test cubes is provided in Figure 9.

We have utilized the optimized settings to print elliptical microchannels in our prototypes. For example, the third prototype has elliptical fluidic channels with a 2.8 mm major axis and 1.8 mm minor axis that lead from the storage reservoirs to distribution chambers (see Figure 4a), while corresponding dimensions of microchannels connecting distribution chambers and detection wells are 3 mm and 2 mm, respectively. 

### 4.2. Postprocessing for Leakage Reduction

Additionally, different post-treatments were tested and evaluated to further minimize leakages. Effectiveness for leakage reduction of the following treatments was tested: exposing the structure to solvent (acetone), coating the structure with a rubber coating (Performix Plasti Dip Multi-Purpose Rubber Coating, Plasti Dip Int.), and dipping the device into heated paraffin wax. Acetone treatment involved dipping the fluidic cartridge in the acetone and soaking it for several seconds. Acetone dissolves the ABS, and the dissolved material resolidifies as acetone evaporates. This process typically allows the dissolved material to fill small trenches between fibers, sealing the gaps. However, the process did not produce consistent results and alternative treatments were selected.

Wax was found to be an effective technique to reduce leakage of 3D-printed fluidic parts. In this method, the fluidic device is dipped into the bath with wax (Paraffin Wax, Laboratory Grade, Carolina Biological Supply Co., Burlington, NC, USA), heated to 80 °C for a few seconds, and while the wax did not have a chance to solidify, the compressed air was blown through the fluidic channels. 

To evaluate the effects of different surface treatments, the following 3D-printed parts were characterized: as-printed untreated ABS surface, printed surface coated with about 1-mm-thick layer of Plasti Dip, printed surface coated with 0.5 mm layer of wax, and printed surfaces after the acetone treatments. The confocal 3D laser scanning microscope (VK-250, Keyence Corp., Osaka, Japan) was used to characterize the surface roughness, and contact angle measurements (MCA-3, Kyowa Interface Ltd., Tokyo, Japan) were also performed.

**Figure A1:**
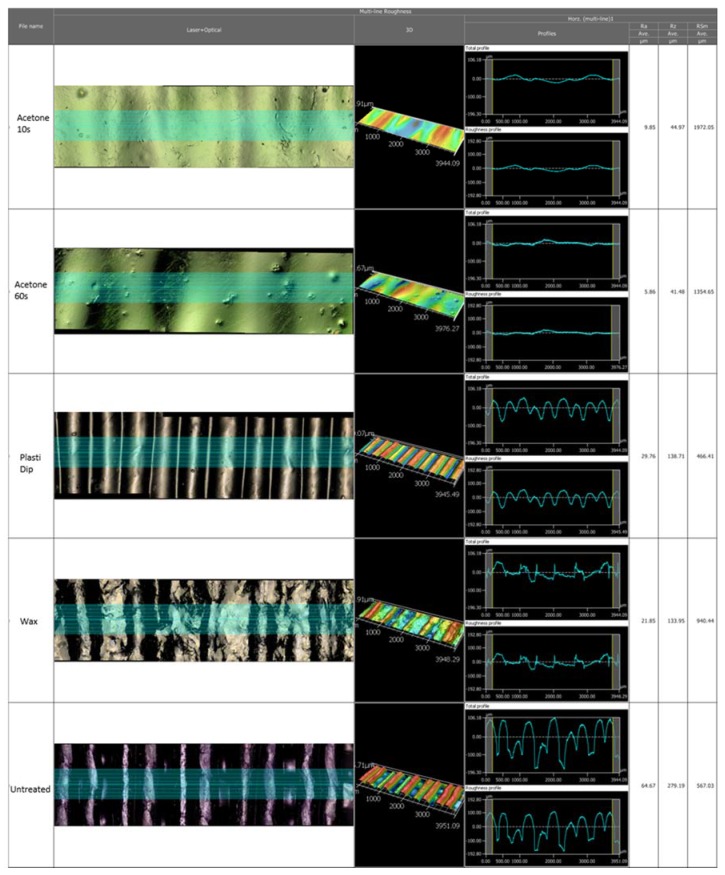
Confocal microscope images and surface roughness of 3D-printed ABS surfaces: Exposed to acetone 10 s, 60 s and air-drying, coated with Plasti Dip, coated with wax, untreated part.

The surface roughness measurements (Figure A1) show an average roughness (Ra) of 64.67 µm for the untreated surface. Plasti Dip coating, wax coating, and acetone exposure for 10 or 60 s decreased this roughness to 29.76, 21.85, 9.85 or 5.86 µm, respectively. 

The contact angle measurements to evaluate hydrophobicity of different surface treatments are shown in Table 4. For each surface, four measurements of the stationary contact angle in different areas of the respective sample were taken (30–60 images for each measurement).

Overall, the wax coating showed the highest hydrophobicity with a contact angle of 102.27° compared to a contact angle of 80.24° for the untreated printed ABS surface. The standard deviation is the highest in the case of the untreated surface, which is assumed to be due to the uneven topography of the part (different results in measurements in a trench versus elevated areas can impact visualization of the contact angle via the camera positioned parallel and on the same level as the evaluated surface). Acetone treatment and wax coating showed the lowest variation in contact angles when compared to untreated and Plasti Dip-coated parts. The average volumes of the droplets were in the range of 15.33 µL to 20.56 µL and are expected to not have significant impact on the measurements. Although the average contact angle after 10 s acetone treatment compared to the untreated part (81.78° versus 80.24°) is slightly higher, 60 s soak in acetone decreases the contact angle drastically to 67.15°. Therefore, the initial increase can be assumed to be within the deviation (especially for the untreated part, the standard deviation is as high at 15.76°).

We have selected to use wax coat inside of the channels and the Plasti Dip coating for the outer surfaces of the fluidic device.

### 4.3. Malaria ELISA Test Results

**Figure 10:**
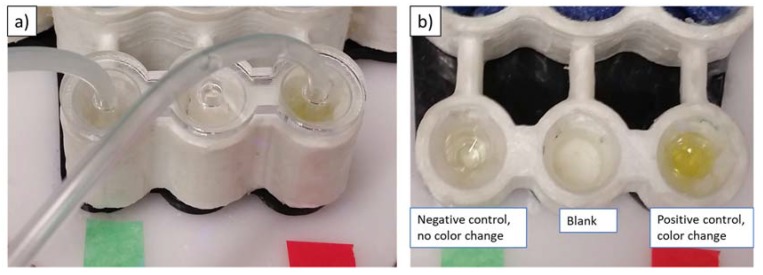
3D-printed device with colorimetric malaria ELISA. (**a**) Laser-cut lid to hold tubes for emptying of wells; (**b**) Colorimetric results after application of stop solution. Left: negative control well, middle: blank well, right: positive control well.

Feasibility of a qualitative readout of the automated bioassay was demonstrated by obtaining a correct color readout for positive and negative controls. Figure 10 shows the automated device after application of the stop solution. Any developed assay with malaria-specific primary antibodies (i.e., positive) would turn yellow in color on addition of the stop solution. The results clearly indicate the negative result (negative control supplied in ELISA kit in the detection well marked green)—that is, no color change, while the positive control (marked red) shows that the color of the solution in the detection well changes to yellow. The tests were repeated several times by replacing the test wells and switching the locations of positive and negative wells to verify repeatability of the experiment. 

## 5. Conclusions

We developed a 3D-printed microfluidic platform that allows for automation of immunoassays and enables portable diagnostics, without the need for trained technicians, that can be employed in limited-resource conditions. The fluidic cartridges can be printed in remote hospitals with inexpensive and increasingly ubiquitous FDM printers to allow on-demand fabrication of automated bioassay platforms. These devices manufactured at local hospitals could then be distributed to health workers and health kiosks in the field. While the disposable fluidic cartridges are printed, the hardware that holds electronics and servomotors is nondisposable and can be used for a variety of bioassays (that would differ by the types of reagents that are loaded, as well as by the sequence of servomotor actuation and timing of various steps). Liquid reagents are loaded into the printed devices prior to usage. Alternatively, reagent-storage options, such as dry storage as well as storage in glass capsules and pouches, can also be utilized for on-board reagent storage [47,48]. The arm movement of the servomotor during the initial actuation could break these pouches or capsules and allow for the reagents to be released. It is possible to pause fabrication to place vials or packages with the reagents into the storage chambers during the printing process. 

The flexible domes, instead of being molded and sealed to the device, could be printed in the same printing process as the microfluidic insert, since a new generation of FDM printers allow for multimaterial printing, including the use of flexible polylactic acid (PLA) [49] and thermoplastic polyurethane (TPU) [50]. 

To the best of our knowledge, this is the first diagnostic device, controlled from an audio port/audio file, presenting an interesting alternative to other types of input into Arduino chips, since replacement of the Arduino program is not required if we want to change the actuation sequence (we just play the new “song”). The demonstrated system, while more complex than lateral immunoassays driven by gravity or capillary forces, allows for automation of complex multi-step bioassays due to presence of servomotors and ability to aspirate fluid from the wells (i.e., washes and dilution steps can be easily integrated). 

While qualitative detection of malaria was demonstrated using positive and negative controls, more studies need to be performed to determine if the developed device can be utilized for quantitative measurements. 

## Figures and Tables

**Table 1:** Comparison of malaria-detection platforms such as rapid diagnostic tests, microscopy, polymerase chain reaction/ loop-mediated isothermal amplification (PCR/LAMP)-based tests, and 3D-printed enzyme-linked immunosorbent assay (ELISA) devices.

Parameter	Rapid Diagnostic Test (RDT)	Microscopy	PCR/LAMP Based in Laboratory	3D-Printed ELISA Lab-on-Chip (LOC)
Sensitivity	Depends on parasite density and species (below 50% for 240–2000 parasites/μL) [12]	Depends on experience of microscopist (parasite counts as low as 5 parasites/μL possible [10], sensitivity 40.9–65.3% [13])	High even for low parasite counts and asymptotic malaria (89.5–93.3%) [14]	Currently only qualitative tests are validated
Time to answer	Fast (<1 h)	Fast (<1 h)	Slow (due to time of transport to the lab)	Medium (<2–3 h)
Cost	Low ($5.22 [15])	Low ($5.53 [15])	High (about C$5–C$25 for PCR plus transportation [16])	Low (less than $10 based on reagent and print costs)
Resources required	None	Trained microscopist, microscope, electricity, clean environment	Laboratory, microplate reader, pipettes, electricity, trained technician	Reusable case with electronics, smartphone (assuming readout is done via smartphone)
Appropriate for low-resource settings	Yes	Maybe	No	Yes
Availability and stability of reagents	No additional reagents required. RDTs are stable for several months to several years [17] (if not exposed to high temperatures and humidity [18])	Staining solutions are stable (although susceptible to mold growth in tropical climate) several weeks to several months [19]	Need to be refrigerated in laboratory, then stable for several months [20]	Need to be refrigerated in laboratory, then stable for several months [20]

**Table 2:** ELISA steps implemented on the 3D-printed automated bioassay platform.

Step	Activity
1	Manual dispensing of 100 µL controls (2 positive, 2 negative, 2 cut-offs, 1 blank) into the wells
2	Incubation for 1 h at 37 °C
3	Washing the wells with approx. 5 × 300 µL, 5 s soak-time each and overflow to the waste/emptying of the wells after each washing step via automated syringe
4	Dispensing of approx. 300 uL malaria conjugate into the wells
5	Incubating the wells at room temperature for 30 min
6	Washing the wells with approx. 5 × 300 µL, 5 s soak-time each and overflow to the waste (prototype 2)/emptying of the wells after each washing step via automated syringe (prototype 3)
7	Dispensing of approx. 100 µL TMB substrate solution in all wells
8	Incubating for exactly 15 min at room temperature, in the dark
9	Dispensing of approx. 100 µL stop solution in all wells
10	Readout of the qualitative result by eye, usage of the smartphone application Color Catcher to record the RGB color codes

**Table 3:** Optimization of print settings for the Flashforge Original Creator using 1.75 mm acrylonitrile butadiene styrene (ABS) filament. Geometry is qualitatively categorized into three types: large deformations (--), small deformations (-), negligible deformations (+).

Test	1	2	3	4	5	6	7	8	9	10
Extrusion temperature (°C)	240	240	**240**	240	240	243	240	240	230	240
Extrusion speed (mm/s)	60	60	**60**	80	80	60	60	80	60	60
Layer height (mm)	0.12	0.14	**0.16**	0.1	0.2	0.1	0.1	0.1	0.2	0.18
Leakage	No	No	**No**	No	Yes	No	No	No	Yes	Yes
Geometry	--	-	**+**	-	+	--	--	--	--	+

**Table 4:** Contact angle measurements of different surface treatments of FDM-printed ABS parts.

Parameter	Untreated ABS	Acetone 10 s	Acetone 60 s	Coat of Wax	Coat of Plasti Dip
Mean contact angle (°)	80.24	81.78	67.15	102.27	72.32
Standard deviation (°)	15.76	2.546	4.246	3.33	10.02
Average droplet volume (µL)	17.41	22.54	15.33	20.56	17.64

## Supplementary Materials

The following are available online at www.mdpi.com/2072-666X/9/1/27/s1, Program Code S1: Prototype with Four Servomotors, Program Code S2: Prototype for the Actuation of up to 8 Servomotors in Two Steps in One.

## Appendix A

**Table A1:** Standard print settings for gears, molds and other non-fluidic elements.

Standard Print Settings
Travelling speed: 90 mm/s
Extrusion speed: 60 mm/s
Heating platform temperature: 115 °C
Extrusion temperature: 240 °C
Number of shells: 2
Layer thickness: 0.2 mm

**Table A2:** An example of a “malaria-song” (with the duration of 9 s for each frequency interval): The 8000 Hz sequences are waiting times outside the programmed frequency-range-activating servomotors.

Frequency in Hz
8000…8000,
1250,
8000…8000,
2250,
8000…8000,
1750,
8000…8000,
3250,
8000…8000,
4250,
8000…8000

**Table A3:** Content of Malaria-Ab kit.

Content of Malaria-Ab Kit
12 break-apart 8-well snap-off strips coated with recombinant Plasmodium antigens (*P. falciparum*, *P. vivax*) in resealable aluminum foil.
1 bottle sample diluent containing 100 mL of buffer for sample dilution; pH 7.2 ± 0.2.
1 bottle stop solution containing 15 mL sulphuric acid, 0.2 mol/L.
1 bottle washing solution containing 50 mL of a 20-fold concentrated buffer (pH 7.2 ± 0.2).
1 bottle malaria conjugate containing 20 mL of peroxidase-labelled antibody to human IgG and IgM.
1 bottle TMB substrate solution containing 15 mL 3,3′,5,5′-tetramethylbenzidine (TMB).
1 bottle malaria positive control containing 2 mL.
1 bottle malaria cut-off control containing 3 mL.
1 bottle malaria negative control containing 2 mL. [20]

**Table A4:** Malaria protocol with wash volumes for automated device.

Recommended Test Protocol
1. Dispensing 100 μL controls and diluted samples into the wells (leave A1 blank).
2. Covering the wells with foil.
3. Incubating the wells for 1 h ± 5 min at 37 °C.
4. Removing the foil, aspirating the content, washing the wells (5 × 350 μL), >5 s soak time, with no overflow; removing fluid by tissue paper.
5. Dispensing 100 μL malaria conjugate in all wells except A1, covering the wells with foil.
6. Incubating the wells at RT for 30 min, with no direct sunlight.
7. Removing the foil, aspirating the content, washing the wells (5 × 350 μL), >5 s soak time, with no overflow; removing fluid by tissue paper.
8. Dispensing 100 μL TMB substrate solution in all wells.
9. Incubating for exact 15 min RT, in the dark.
10. Dispensing 100 μL stop solution in all wells, in the same order and rate as the TMB substrate solution.
11. Measuring the absorbance of the specimen at 450/620 nm within 30 min after dispensing the stop solution.
