# Supplementary material for: Fabrication of a Lab-on-Chip Device Using Material Extrusion (3D Printing) and Demonstration via Malaria-Ab ELISA
Source: Micromachines (Basel). 2018 Jan 14;9(1):27. doi: 10.3390/mi9010027 (PMC6187338; doi:10.3390/mi9010027)
Supplement: Supplementary file 1 [file micromachines-09-00027-s001.pdf]

Supplementary Materials: Fabrication of a Lab-on-Chip Device Using Material Extrusion (3D Printing) and Demonstration via Malaria-Ab ELISA

Maria Bauer and Lawrence Kulinsky *

1. Program Code for a Prototype with Four Servomotors

```
/*------------------------------------------------------------------------
------------------------------------------------------------------------*/
#ifndef F_CPU
#define F_CPU 16000000
#endif
#include <avr/io.h>
#include <avr/interrupt.h>
volatile uint16_t calculated_frequency = 0;
volatile uint16_t zero_cross_detection_counter = 0;
volatile uint8_t ledblink = 0;
//------------------------------------------------------------------------
//start of the main programm
int main(void)
{
int8_t pwm1 = 0;
int8_t pwm2 = 0;
int8_t pwm3 = 0;
int8_t pwm4 = 0;
// Set Outputs for LEDs
DDRB |= (1 << DDB5) | (1 << DDB4);
// Set Outputs for Timer 1
DDRB |= (1 << DDB2) | (1 << DDB1);
// Set Outputs for Timer 2
DDRB |= (1 << DDB3);
DDRD |= (1 << DDD3);
// Set Timer 0 for 100Hz
TCCR0A |= (0<<COM0A1) | (0<<COM0A0) | (0<<COM0B1) | (0<<COM0B0) | (0<<WGM01) |
(0<<WGM00);
TCCR0B |= (1<<CS02) | (0<<CS01) | (1<<CS00);
TIMSK0 |= (1<<OCIE0A);
OCR0A = 156;
// Set Timer 1 for for 61Hz with fast PWM
TCCR1A |= (1<<COM1A1) | (0<<COM1A0) | (1<<COM1B1) | (0<<COM1B0) | (0<<WGM11) |
(1<<WGM10);
TCCR1B |= (0<<WGM13) | (1<<WGM12) | (1<<CS12) | (0<<CS11) | (1<<CS10);
OCR1A = 23;// Min: 13Center: 23Max: 33
```

```
OCR1B = 23;
// Set Timer 2 for 61Hz with fast PWM
TCCR2A |= (1<<COM2A1) | (0<<COM2A0) | (1<<COM2B1) | (0<<COM2B0) | (1<<WGM21) |
(1<<WGM20);
TCCR2B |= (1<<CS22) | (1<<CS21) | (1<<CS20);
OCR2A = 23; // Min: 13Center: 23 Max: 33
OCR2B = 23;
// Analog Comparator
ACSR |= (1<<ACIE) | (0<<ACIS1) | (0<<ACIS0);
sei();
while(1)
{
pwm1 = 0;
pwm2 = 0;
pwm3 = 0;
pwm4 = 0;
if ((calculated_frequency>500) & (calculated_frequency<1000))
{
pwm1 = -8.5; //Fullway 1
}
if ((calculated_frequency>1100) & (calculated_frequency<1400))
{
pwm1 = -7.5; //Halfway 1
}
if ((calculated_frequency>1500) & (calculated_frequency<2000))
{
pwm2 = 6; //Fullway 2
}
if ((calculated_frequency>2100) & (calculated_frequency<2400))
{
pwm2 = 5; //Halfway 2
}
if ((calculated_frequency>2500) & (calculated_frequency<3000))
{
pwm3 = -5.5; //Fullway 3
}
if ((calculated_frequency>3100) & (calculated_frequency<3400))
{
pwm3 = -5; //Halfway 3
}
if ((calculated_frequency>3500) & (calculated_frequency<4000))
{
pwm4 = 5.8; //Fullway 4
```

```
}
if ((calculated_frequency>4100) & (calculated_frequency<4400))
{
pwm4 = 5; //Halfway 4
}
OCR1A = 23 + pwm1;
OCR1B = 23 + pwm2;
OCR2A = 23 + pwm3;
OCR2B = 23 + pwm4;
}
return(0);
}
ISR (ANALOG_COMP_vect)
{
ACSR &= ~(1<<ACIE);
// Delay about 50µs
uint8_t test = 0;
for (test=0; test<115; test++)
{
PORTB ^= (1<<4);
}
ACSR |= (1<<ACIE);
zero_cross_detection_counter++;
}
ISR(TIMER0_COMPA_vect)
{
TCNT0 = 0;
ledblink++;
if (ledblink > 99)
{
ledblink = 0;
PORTB ^= (1<<5);
}
calculated_frequency = (zero_cross_detection_counter*100)/2;
zero_cross_detection_counter = 0;
}
```

2. Program Code for Prototype for the Actuation of up to 8 Servomotors in Two Steps in One Direction

```
/*---------------------------------------------------------------------------
---------------------------------------------------------------------------*/
#ifndef F_CPU
#define F_CPU 16000000
#endif
#include <avr/io.h>
#include <util/delay.h>
#include <avr/interrupt.h>
volatile uint8_t pwm_isr_count = 0;
volatile uint8_t pwm_array[8] = {30,30,30,30,30,30,30,30};
volatile uint8_t isr_i;
volatile uint16_t calculated_frequency = 0;
volatile uint16_t zero_cross_detection_counter = 0;
volatile uint8_t ledblink = 0;
volatile uint8_t temp_a = 0;
volatile uint8_t temp_b = 0;
volatile uint8_t temp_d = 0;
volatile uint16_t freqblink = 0;
//---------------------------------------------------------------------------
//Start of main program
int main(void)
{
// Set Outputs for LEDs
DDRB |= (1 << DDB5) | (1 << DDB2) | (1 << DDB1) | (1 << DDB0);
// Set Outputs for PWM
DDRC |= (1 << DDC5) | (1 << DDC4) | (1 << DDC3) | (1 << DDC2) | (1 << DDC1) | (1 << DDC0);
DDRD |= (1 << DDD5) | (1 << DDD4);
// Set Timer 0 for 100Hz
TCCR0A |= (0<<COM0A1) | (0<<COM0A0) | (0<<COM0B1) | (0<<COM0B0) | (0<<WGM01) |
(0<<WGM00);
TCCR0B |= (1<<CS02) | (0<<CS01) | (1<<CS00);
TIMSK0 |= (1<<OCIE0A);
OCR0A = 156;
// Set Timer 1 for PWM
TCCR1A = 0;
TCCR1B |= (1<<WGM12);
TCCR1B |= (0<<CS12) | (1<<CS11) | (0<<CS10);
OCR1A = 100; // 4000 = 2ms // 100 = 50us
TIMSK1 |= (1<<OCIE1A);
// Analog Comparator
ACSR |= (1<<ACIE) | (0<<ACIS1) | (0<<ACIS0);
```

```
sei();
uint8_t servo_1_steps = 0;
uint8_t servo_2_steps = 0;
uint8_t servo_3_steps = 0;
uint8_t servo_4_steps = 0; // Servos 5-8 are not being used for Malaria kit
while(1)
{
PORTB ^= (1<<PB0);
uint8_t i;
//all cannels to the mid position
for (i=1; i<8; i++)
{
pwm_array[i] = 30; // 30*50µs = mid position = 1.5ms
}
if ((calculated_frequency>1000) & (calculated_frequency<1500))
{
//when frequency first reaches 500-1000 Hz, servo_1_steps is raised to 1
if (servo_1_steps == 0)
{
servo_1_steps = 1;
}
}
if ((calculated_frequency>1500) & (calculated_frequency<2000))
{
if (servo_1_steps == 1)
{
servo_1_steps = 2;
}
}
if (servo_1_steps == 0)
{
pwm_array[1] = 25;
}
if (servo_1_steps == 1)
{
pwm_array[1] = 30;
}
if (servo_1_steps == 2)
{
pwm_array[1] = 35;
}
//Servo 2
if ((calculated_frequency>2000) & (calculated_frequency<2500))
```

```
{
//when frequency first reaches 500-1000 Hz, servo_2_steps is raised to 1
if (servo_2_steps == 0)
{
servo_2_steps = 1;
}
}
if ((calculated_frequency>2500) & (calculated_frequency<3000))
{
if (servo_2_steps == 1)
{
servo_2_steps = 2;
}
}
if (servo_2_steps == 0)
{
pwm_array[2] = 25;
}
if (servo_2_steps == 1)
{
pwm_array[2] = 30;
}
if (servo_2_steps == 2)
{
pwm_array[2] = 35;
}
// Servo 3
if ((calculated_frequency>3000) & (calculated_frequency<3500))
{
//when frequency first reaches 500-1000 Hz, servo_3_steps is raised to 1
if (servo_3_steps == 0)
{
servo_3_steps = 1;
}
}
if ((calculated_frequency>3500) & (calculated_frequency<4000))
{
if (servo_3_steps == 1)
{
servo_3_steps = 2;
}
}
if (servo_3_steps == 0)
```

```
{
pwm_array[3] = 25;
}
if (servo_3_steps == 1)
{
pwm_array[3] = 30;
}
if (servo_3_steps == 2)
{
pwm_array[3] = 35;
}
//Servo 4
if ((calculated_frequency>4000) & (calculated_frequency<4500))
{
//when frequency first reaches 500-1000 Hz, servo_4_steps is raised to 1
if (servo_4_steps == 0)
{
servo_4_steps = 1;
}
}
if ((calculated_frequency>4500) & (calculated_frequency<5000))
{
if (servo_4_steps == 1)
{
servo_4_steps = 2;
}
}
if (servo_4_steps == 0)
{
pwm_array[4] = 25;
}
if (servo_4_steps == 1)
{
pwm_array[4] = -30;
}
if (servo_4_steps == 2)
{
pwm_array[4] = -35;
}
//Reset of Servos
if ((calculated_frequency>10) & (calculated_frequency<500))
{ servo_4_steps = 0;
servo_1_steps = 0;
```

```
servo_2_steps = 0;
servo_3_steps = 0;
}
if ((calculated_frequency>5500) & (calculated_frequency<6000))
{
pwm_array[5] = 20;
}
if ((calculated_frequency>6500) & (calculated_frequency<7000))
{
pwm_array[6] = 20;
}
if ((calculated_frequency>7500) & (calculated_frequency<8000))
{
pwm_array[7] = 20;
}
}
OCR1A = 100;
TCNT1 = 0;
TIMSK1 |= (1<<OCIE1A);
_delay_ms(20);
return(0);
}
//run, when speakers signal goes through 0
// ISR = Interrupt Service Routine
ISR (ANALOG_COMP_vect)
{
//ISR off to avoid double-run
ACSR &= ~(1<<ACIE);
freqblink++;
if (freqblink > 2000)
{
freqblink = 0;
PORTB ^= (1<<PB1); // PB1 = yellow to opposite signal
}
// Delay about 50us
_delay_us(50);
// uint8_t test = 0;
// for(test=0; test<115; test++)
// {
// PORTB ^= (1<<PB4);
// }
// ISR on again
ACSR |= (1<<ACIE);
```

```
zero_cross_detection_counter++;
}
// Zeit ISR 100 times per second
ISR(TIMER0_COMPA_vect)
{
// start time again
TCNT0 = 0;
ledblink++;
if (ledblink > 99)
{
ledblink = 0;
PORTB ^= (1<<PB2); // blink red LED once a second
}
calculated_frequency = (zero_cross_detection_counter*100)/2;
zero_cross_detection_counter = 0;
}
// ISR every 50µs
ISR (TIMER1_COMPA_vect)
{
// TCNT1 = 0 and OCR1A = 2000, or only OCR1A += x;
//PORTC ^= (1<<0);
//PORTD |= (1<<5);
// first time then all servos to high
// bitmanipulation
if (pwm_isr_count == 0)
{
PORTC |= (1<<PC5) | (1<<PC4) | (1<<PC3) | (1<<PC2) | (1<<PC1) | (1<<PC0);
PORTD |= (1<<PD4) | (1<<PD5);
}
else
{
temp_b = 0; // values from port c
temp_d = 0;
temp_a = 0; // dummy, only used to reach same runtime
for (isr_i=0; isr_i<6; isr_i++)
{
// when counter higher than value in array, then save in temp_b that the pwm channel has to be
lowered
if (pwm_isr_count>=pwm_array[isr_i])
{
temp_b |= (1<<isr_i);
//PORTC &= ~(1<<isr_i);
}
```

```
// else
// {
// temp_a |= (1<<isr_i);
// }
}
for (isr_i=6; isr_i<8; isr_i++)
{
if (pwm_isr_count>=pwm_array[isr_i])
{
temp_d |= (1<<(isr_i-2));
//PORTD &= ~(1<<(isr_i-2));
}
// else
// {
// temp_a |= (1<<isr_i);
// }
}
// ports = saved values = which servos have to be lowered
PORTC &= ~temp_b;
PORTD &= ~temp_d;
}
// count up timevariable
pwm_isr_count++;
// 40*50µs = 2000µs = 2ms = maximum lenght of pwm
// 1ms = left
// 1,5ms = middle
// 2ms = right (or left)
if (pwm_isr_count > 40)
{
// start new
TIMSK1 = 0;
pwm_isr_count = 0;
}
//PORTD &= ~(1<<5);
    }
```
